# What the Newer Therapeutic Procedures Have Done for Neurology1Read before the Section on Medicine of the Buffalo Academy of Medicine, October 10, 1893.

**Published:** 1893-11

**Authors:** William C. Krauss

**Affiliations:** 382 Virginia Street; Buffalo, N. Y.


### WHAT THE NEWER THERAPEUTIC PROCEDURES HAVE DONE FOR NEUROLOGY.[1]

By WILLIAM C. KRAUSS, M. D., Buffalo, N. Y.

THE epoch in which we live may well be called the sky-rocket period of the XIX. century. Men, like methods, approach their zenith with an increasing roar and gusto, burst into sparkling brilliancy, and as suddenly fade and fall to the ground with a dull and heavy thud. What was yesterday a seemingly brilliant success becomes today a glittering failure, and the shores of time are laden with the wrecks of "wonderful discoveries."

Hypnotism, suspension, and the method of Brown-Séquard have each enjoyed their sky-rocket experience, and the impressions which they left after spending their force is what we propose to study tonight.

1. Read before the Section on Medicine of the Buffalo Academy of Medicine, October 10, 1893.

The first reports of the method of Brown-Séquard read like a fairy tale, and the "Elixir of Life," so-called, seemed to be the magic fluid that philosophers had sought to compound for centuries back. No doubt, Brown-Séquard was perfectly honest in the thought that he had invented a method unsurpassed and hitherto undiscovered, but on searching the alcoves of the National Library of Paris several brochures have been found, written by Dr. Mizauld, which contain much of interest if not of actual worth. This physician lived in Paris in the XVI. century, and the following passage must certainly establish for him a certain right to priority in favor of a method which, sleeping for several centuries, was re-awakened by Brown-Séquard. He says: "If the genital organs of a red bull be bruised in a mortar and taken by a woman in some wine or soup, it will give her a disgust for love, while, to the contrary, the same beverage taken by a debilitated man will re-awaken his amorous desires." Brown-Séquard said nothing any more explicit in his well-known communication to the Société de Biologie of Paris, on June 1, 1889.

It seems that Brown-Séquard had been at work on this project for many years, for, in 1869, he expressed a belief that if it were possible to inject spermatic fluid into the veins of old men they would experience a rejuvenation—sexually, mentally, and physically. After repeated experiments upon rabbits, dogs, and guinea-pigs, he, in a true scientific spirit, injected some of the testicular fluid into his system, and his experiences and results form the most interesting part of his memorable communication to this learned society:

> The author of this communication, now seventy-two years old, has for the past twelve years watched his physical powers slowly and continually decline. The laboratory work has become laborious and heavy, and after each meal I have been obliged to take a short nap. After the third injection a complete change took place. The work in the laboratory has become agreeable, not the least fatiguing, and after three and a half hours of such work I have been able to edit a memoir. The dynamometer showed an increase of 6.7 kilogrammes, the bowels regained their former activity, and, in short, I have regained all that I have lost.

For some time the most enthusiastic reports were received, especially by Hammond and Loomis in this country, D'Arsonval, Villeneuve, Mairet, Gley, Hirschberg, and Egasse in France, Marro and Rivano, Ventro, Copriati, and Mosso in Italy, Owspenski in

Russia, and a host of other observers, each one eager to land his results on the ground floor. The diseases treated were general debility, locomotor ataxia, insanity, impotence, cholera, tuberculosis, cardiac weakness, nervous dyspepsia, lumbago, hemiplegia, myalgia, neurasthenia, etc. All of these affections have either been cured or else greatly benefited by injections of testicular juice, that is, in 1889 and 1890 especially.

Gradually the reports became less numerous and less encouraging, save those which came from the master himself and some of his former pupils. Perhaps the greatest check to this movement was the fact that Charcot and his pupils refrained from using these injections, or, at least to my knowledge, never gave it their sanction. Negel, of Jassy, France, has reported recently his experience with this fluid, and in a large number of cases treated, of various affections of the nervous system, failed to obtain any results whatever. Pulawski, of Warsaw, Russia, made a series of experiments upon twelve cases, and came to the following conclusions :

Local pain and abscess formation ; fever with chills ; no specific action ; subjective and positive amelioration were dependent upon suggestion.

Copriati studied the effect of testicular juice in four cases of insanity, and found that it had no dynamogenic influence on the nerve centers, its effects being limited to temporary stimulation of the nervous system. The unkindest cut of all was the report of Féré, one of the ablest of French neurologists, who, at the request of D'Arsonval, gave the method a thorough trial at the Bicêtre hospital. In his communication to the Société de Biologie, just four years after Brown-Séquard's, he, in unmistakable language, disapproves of the method and cites nine cases which had been under treatment. No favorable result was obtained in any case ; on the contrary, the injections seemed to act as a depressant. Ovarian juice has, according to Brown-Séquard, given similar though less marked results.

Spermine is the name of another fluid extract derived from Brown-Séquard's testicular juice by Poehl. Its action seems to be similar to the testicular juice, acting upon the motor areas of the cerebro-spinal axis, increasing the strength of the arms and legs, regulating the sexual, urinary, and digestive functions, and in improvement of the general sensibility.

Brown-Séquard's method today is not used by neurologists either in America or Europe, but is still being experimented with by its champion and his pupils apparently with good results in a certain class of functional nervous diseases.

Following closely upon this method of treatment Gley, decided to inject the juice of thyroid glands in dogs thus deprived of these glands, and, instead of dying, they recovered without any serious difficulties. In the human family it has been found that after removal of the thyroid gland through disease, that a certain train of symptoms will develop, which have received the name of myxedema, a disease characterized by swelling of the face, body, and extremities, loss of hair, sub-normal temperature, etc. Horsley attempted to transplant the thyroid gland of animals to these patients, and met with partial success. Dr. Murray, of Newcastle, England, then injected hypodermically a glycerine extract of thyroid gland into patients suffering with myxedema, and his efforts were rewarded with beneficial results. Brown-Séquard and D'Arsonval were conducting similar experiments about the same time with equally good success. It was found, however, that the injection of this substance was followed in many cases by pain, inflammation, and abscess formation. To overcome these hindrances, Fox of Plymouth and Mackenzie advised and practised the treatment of myxedema by feeding with sheeps' thyroid glands, and the results seemed to be in every way satisfactory.

The writer has had a little experience in treating two cases of myxedema, but he has been unable to attain anything like the results claimed by the English and French writers. In fact, his experience has been negative, and not even obtaining temporary improvement.

MacAlister, of England, has treated cases of pseudo-hypertrophic paralysis with injections of thymus gland extract ; also a case of lymphadenoma with a mixture of red and yellow marrow, with seemingly good results.

Dieulafoy, of Paris, has injected extracts of the cortical portion of the kidney into patients suffering with Bright's disease. He proposes the name Nephrine for this particular fluid.

Comby and Dieulafoy have also injected the extract of pancreas in cases of diabetes with temporary good results.

Following the footsteps of Constantin Paul, of Paris, an American experimenter has injected a large list of specific agents into our *vis medicatrix.* Cerebrine (Hammond) and cerebrin (Parke, Davis & Co.), medulline, cardine, ovarine, testine, musculine, etc., are the newly-coined words which describe these preparations. I need not tell you what has been claimed for these fluids, for, no doubt, you have all read the paper extolling their virtues and efficacy, published in nearly every medical journal of America.

I have tried to give cerebrin a good, fair trial, and have used it in two cases of locomotor ataxia, two of epilepsy, two of neurasthenia, and one of general debility. Not a single one reported improvement; not even did a reaction set in. The only visible effect was the disappearance of the patients.

Archie Stockwell, in an interesting paper published in the *Medical News*, August 26, 1893, describes his experience with the two rival cerebrines, and a mixture of borax, glycerine, and water. He comes to the conclusion that these three preparations are equally efficacious, or rather equally inert for good or evil. Negel, of Jassy, also experimented with cerebrine without any appreciable results. Negative results, when reported, have a greater significance than successful results, because many observers are unwilling to have their failures paraded in the medical press; besides, many editors are averse to publishing articles detrimental to their advertisers.

My conclusions, then, in regard to the animal extracts, are: That since recent experiments fail to corroborate the results obtained immediately after the introduction of Brown-Séquard's method, the whole matter must be left open for further investigation. Secondly, that many of the results obtained were through suggestion and auto-suggestion, and that no specific action has been discovered.

In regard to the treatment of myxedema, although my results were negative, I believe that there is some virtue in the various methods of introducing thyroid glands into these patients, but the disease must be of recent standing and the patient not advanced in years.

As to the injection of the i-n-e compounds, I believe that it is all rot. I cannot be convinced that injections of musculine will cure an atrophied muscle, due to destruction of the ganglion cells of the ventral horns of the spinal cord; or that medulline will cure a sclerosed cord, the most common form of cord disease; or that cerebrine will cure apoplexy cerebralis, perhaps the most common form of brain disease.

Just recently there has appeared a work by Chèron, of Paris, who writes pointedly on this subject. He says:

All liquids, when introduced under the skin, produce identical effects, provided they are not toxic and have no specific toxic action.

They increase arterial tension, and, in the diseases in which these fluids have been used, a degree of hypo-tension has existed, which being relieved by injections, temporary results have followed.

SUSPENSION.

Raymond, a pupil of Charcot, while studying the Russian University system in 1888, discovered Motchouskowski, of Odessa, suspending his cases of locomotor ataxia with beneficial results. Motchouskowski had himself discovered this method by accident in 1883, and, although published at that time, it had been entirely unheeded and forgotten. It was found that the lancinating pains, vesical and sexual disorders, eye symptoms, and the ataxic gait, would yield when all other remedies had failed. On returning to Paris, this method was tried secretly by the internes at the Salpêtriere, and after obtaining satisfactory results, was divulged to Charcot, who at once instituted a thorough trial. I had the pleasure of being in Paris at this time, and saw and examined many of the patients thus treated. New treatment gives new results, and many of the old stagers declared they were much improved and getting well. Charcot never claimed that suspension would cure locomotor ataxia, or any other organic disease of the cord, but the report gained ground that it would cure permanently, and the method soon fell in disrepute. All that was claimed for it was that it would relieve some of the terrible symptoms, and now, five years after its re-introduction to the profession, let us see what is still claimed for it.

Von Bechterew, perhaps the foremost Russian neurologist, says in *Neurologisches Centralblatt*, September 15, 1893:

> The suspension treatment has continued to exert a favorable influence on all cases thus far treated; particularly beneficial has it been in locomotor ataxia, spinal syphilis, transverse and central myelitis, compression myelitis, and compression of the spine. In some of these cases it has produced seemingly permanent good results, as nearly a year has elapsed and the patients still enjoy good health.

Writing, on April 1, 1893, in the same journal, he recounts the favorable influence it has upon the optic nerve in spinal-cord affctions. Sprymon has had similar good results in locomotor ataxia and myelitis. Benedikt, of Vienna, another leader of neurological thought, has had, in a number of severe cases of tabes, apparently astonishing results. Patients who were quite powerless to walk or stand were enabled to take long promenades with, and sometimes without, a cane. Neuralgic attacks seemed to be more often influenced by this method than any other train of symptoms.

Bonjour, of Zurich, in treating eighteen cases, thirteen of which were locomotor ataxia, obtained excellent results in the alleviation of some of the symptoms in every case. Duncan, of Glasgow, reported recently a case of locomotor ataxia with considerable improvement. Bogroff, of Paris, likewise reports success in his cases. Gray, in his recent work on nervous diseases, one of the best from a therapeutic standpoint, says:

> Suspension, indeed, is a new fad that has certainly effected a temporary improvement in all the symptoms of some cases, often to a wonderful degree. Thus, in one case of my own, in the last stages of the disease, this remedy was tried as a last resort, and, incredible as it may seem, the patient, after two suspensions, got out of bed, which he had not left for weeks, and walked down several flights of stairs.

Other favorable results have been obtained by Rumpf, of Marburg; Althaus, of London; Mendel, of Berlin, and a host of other men high in neurological circles. Hirt, in his admirable text-book, recently translated into English, has had a somewhat monotonous experience. He treated 114 cases of locomotor ataxia (eighty-nine men and twenty-five women) by suspension. "In no single instance," says he, "was I able to note any marked or lasting improvement, and in no case was either the general condition of the patient, or the course of the disease, influenced for the better; nay, even in the individual symptoms, no decided improvement could be perceived." This experience is rather surprising, because, coming from such a keen observer, he certainly would have detected results had they been forthcoming.

My experience with suspension has been very satisfactory, partly because I did not expect to see my cases cured in a few days, and partly because I would advocate this mode of treatment as a last resort, and was content with any relief, however slight it might have been. I treated three cases of locomotor ataxia, two of hemiplegia, three of railway spine, two of neurasthenia, and one of multiple sclerosis of the cord. One case of locomotor ataxia, a prominent business man in this city, came to me with all the characteristic symptoms of this disease, such as Romberg's, Westphal's, and Argyll Robertson's symptoms, ptosis, and strabismus, lancinating pains, ataxic gait, vesical and sexual disorders, stomach crises, etc. Surely a typical case of tabes. His treatment consisted of suspension three times weekly and spinal galvanism.

After five months of such treatment, I found that the tabetic symptoms had all disappeared; save the myosis. Even the tendon reflexes had returned, though not to their normal intensity. Today he is at his work, thoroughly convinced that he has been cured of locomotor ataxia. Occasionally he comes to be suspended, and on each occasion I find his condition improving. I would not dare claim that he has been cured or permanently benefited, because I cannot believe that a spinal cord once sclerosed can be cleared up, any more than a hobnailed liver can be repaired to its former usefulness. The other two cases of locomotor ataxia were temporarily benefited, especially the gait and pains. One case of hemiplegia recovered splendidly, surprising even herself; the other case died before the results came! The case of multiple sclerosis grew worse, if anything, while the cases of railway spine and neurasthenia have done well, and, supplemented by other treatment, have recovered.

From all these reports, with the exception of Hirt's, we are justified in saying that suspension has done all that was promised for it—sometimes doing more, sometimes less. When we consider how exasperating are some of the symptoms of locomotor ataxia, the least palliation that this treatment affords should be gladly embraced and thanks returned. I doubt whether it will ever disappear entirely as a therapeutic procedure in the treatment of spinal cord diseases.

### HYPNOTISM.

Hypnotism and suggestion, another method which has at different times claimed the attention of experimenters, but not until recently has it been considered a therapeutic agent. Animal magnetism of Mesmer, Hypnotism of Braid, and Suggestion of Charcot is a brief history of the development of this strange phenomenon. Each of these experimenters has done much to unravel the mysteries surrounding this agent, but to Charcot must be credited the honor of snatching it from chicanery and giving it a certain respectability. Liebault, Liègois, and Bernheim must be commended for their zeal and interest, while Luys has plainly carried it beyond the limit of science and truth. Time will not permit to enter into a discussion of the various stages, the different methods, or into the points of difference between the Charcot and Nancy schools, but merely to indicate its applicability and the results that may be expected. I need not recall to you the wonderful results obtained by observers, the world over, during the years 1886–1890; how long-standing chronic diseases of the brain and cord disappeared like the dew, and how in it was found the panacea of human ills. These much desired qualities were, however, of very short duration, for the crucial tests were soon applied, and hypnotism and suggestion quickly found their proper sphere.

Hypnotic suggestibility depends first upon the presence of extreme instability of the cellular nervous elements, and secondly, upon a weak power of inhibition or control of the activity of these elements. Persons of a low order of intellect are not favorable subjects for hypnosis; neither are persons of a strong individuality, nor the insane. The class of cases most favorable for hypnotic treatment are the hysterical; first, because they can be easily hypnotized, and secondly, because the disease requires a treatment which appeals directly to the perverted action of the cerebral centers. It surely is not indicated for exhibition purposes, or for the treatment of any nervous disease or state, unless all other remedies have been exhausted. Even in hysteria this holds equally true. Binswanger, of Jena, in reviewing the literature on the use of hypnotism in the treatment of the insane, finds that the best results were obtained in hysterical insanity, but in a number of cases of melancholia, and chronic alcoholism, hypnotic suggestion had marked success. Berillon, in treating 300 cases, one-third of which were hysterical, had good results in almost all from the use of hypnotism. Collins, of New York, Dujardin-Beaumetz, of Paris, and many others, have had good results in hysterical conditions, and uphold the Charcot doctrine. Almost every functional nervous disorder, and many of the organic diseases of the nervous system, have been benefited by hypnotism. My cases were all of hysteria, and generally of the dull phlegmatic temperament. Neurasthenia, and the excited states, are rarely ever benefited. I agree with Berillon that hypnotism is indicated (1) in the spasmodic attacks of grave hysteria and the paralysis following, (2) in mono-symptomatic hysteria, (3) in ordinary hysteria, and in (4) hysterical insanity.

This subject is of such recent discussion in medical literature, and the diseases treated so various, that I will refrain from taking any more of your valuable time. In conclusion, I may repeat that hypnotism is of the greatest therapeutic importance in some cases of hysteria, but that its use should be delayed until it is absolutely demanded.

382 VIRGINIA STREET.

